# Contribution of Non-canonical Cortisol Actions in the Early Modulation of Glucose Metabolism of Gilthead Sea Bream (*Sparus aurata*)

**DOI:** 10.3389/fendo.2019.00779

**Published:** 2019-11-12

**Authors:** Jorge E. Aedo, Ignacio Ruiz-Jarabo, Gonzalo Martínez-Rodríguez, Sebastián Boltaña, Alfredo Molina, Juan A. Valdés, Juan M. Mancera

**Affiliations:** ^1^Facultad de Ciencias de la Vida, Universidad Andrés Bello, Santiago, Chile; ^2^Interdisciplinary Center for Aquaculture Research (INCAR), Universidad de Concepción, Concepción, Chile; ^3^Department of Biology, Faculty of Marine and Environmental Sciences, Instituto Universitario de Investigación Marina (INMAR), Campus de Excelencia Internacional del Mar (CEI-MAR), University of Cádiz, Cádiz, Spain; ^4^Department of Marine Biology and Aquaculture, Instituto de Ciencias Marinas de Andalucía (ICMAN-CSIC), Puerto Real, Spain

**Keywords:** cortisol, gene expression, glucose metabolism, membrane-initiated cortisol action, *Sparus aurata*, stress response

## Abstract

Teleost fish are exposed to diverse stressors in farming and wildlife conditions during their lifespan. Cortisol is the main glucocorticoid hormone involved in the regulation of their metabolic acclimation under physiological stressful conditions. In this context, increased plasma cortisol is associated with energy substrate mobilization from metabolic tissues, such as liver and skeletal muscle, to rapidly obtain energy and cope with stress. The metabolic actions of cortisol have primarily been attributed to its genomic/classic action mechanism involving the interaction with intracellular receptors, and regulation of stress-responsive genes. However, cortisol can also interact with membrane components to activate rapid signaling pathways. In this work, using the teleost fish gilthead sea bream (*Sparus aurata*) as a model, we evaluated the effects of membrane-initiated cortisol actions on the early modulation of glucose metabolism. For this purpose, *S. aurata* juveniles were intraperitoneally administrated with cortisol and with its membrane impermeable analog, cortisol-BSA. After 1 and 6 h of each treatment, plasma cortisol levels were measured, together with glucose, glycogen and lactate in plasma, liver and skeletal muscle. Transcript levels of corticosteroids receptors (*gr1, gr2*, and *mr*) and key gluconeogenesis (*g6pc* and *pepck*)- and glycolysis (*pgam1* and *aldo*) related genes in the liver were also measured. Cortisol and cortisol-BSA administration increased plasma cortisol levels in *S. aurata* 1 h after administration. Plasma glucose levels enhanced 6 h after each treatment. Hepatic glycogen content decreased in the liver at 1 h of both cortisol and cortisol-BSA administration, while increased at 6 h due to cortisol but not in response to cortisol-BSA. Expression of *gr1, g6pc, pgam1*, and *aldo* were preferentially increased by cortisol-BSA in the liver. Taking all these results in consideration, we suggest that non-canonical cortisol mechanisms contribute to the regulation of the early glucose metabolism responses to stress in *S. aurata*.

## Introduction

Fish are exposed to diverse stressful conditions in farming and wildlife during their lifespan (1, 2). Physiological acclimation to stress is essential to mobilize energy reserves (primarily glucose) and help the animal to overcome the threat (3). The physiological response to stress is initiated by the activation of the hypothalamic–pituitary–adrenal (HPA) and the hypothalamic-pituitary-interrenal (HPI) axis, secreting catecholamines and glucocorticoids, respectively (1). Both hormones are critical to coordinate multiples steps of the physiological and metabolic responses to maintain homeostasis (1, 4, 5).

Cortisol is the main glucocorticoid hormone involved in the regulation of the metabolic/physiological adaptation under stress conditions in teleost fish (4, 6). During the first phases of the stress response, increases of plasma cortisol levels produce the enhancement in glucose mobilization to vital organs mainly through the induction of hepatic gluconeogenesis as well as the inhibition of glycolysis and glucose uptake of peripheral organs (4, 7). In this context, it is well-known that cortisol is directly involved in the expression of key gluconeogenesis-related genes such as phosphoenolpyruvate carboxykinase (*pepck)* (8). Conversely, cortisol action on the transcriptional regulation of skeletal muscle glucose homeostasis is less understood and is mainly associated to the permissive-role for catecholamines induced glycogenolysis, as well as the modulation of glycolysis related genes such as pyruvate dehydrogenase kinase (*pdk*) (7). Accordingly, cortisol has an important role in the transcriptional modulation of glucose metabolism in fish (9).

The mechanism of cortisol action is associated with its genomic/classic signaling pathways involving the interaction with intracellular receptors and the modulation of stress responsive genes (9). Owing to the lipophilic nature of cortisol, this hormone is capable of crossing inside the cell and interact with its intracellular mineralocorticoid (MR) and glucocorticoid (GR) receptors (9, 10). The complex cortisol-receptor acts as a transcriptional factor interacting with glucocorticoid response elements (GRE) localized in stress-responsive genes. In this context, the identification of GRE located in gluconeogenesis and glycolysis related genes (e.g., *pepck* and *g6pc*) supports the idea of a direct regulation of the glucose metabolism by cortisol through transcriptional mechanisms (11, 12).

In addition to classical intracellular receptor binding, there is evidence that cortisol and other steroid hormones can also interact with membrane components activating rapid signaling pathways (13–15). This novel mechanism for cortisol is known as a non-genomic action and it has mainly been characterized using steroids analogs coupled to hydrophilic molecules such as bovine serum albumin (BSA) (13, 16). The complex glucocorticoid-BSA is an exclusive inductor of membrane-initiated effects and has been successfully used to discriminate the novel glucocorticoid actions in both *in vitro* and *in vivo* models (16–19). In fish models, the non-genomic cortisol actions have been associated with the suppression of phagocytosis in the freshwater teleost *Channa punctatus* (17) and with the increased plasma membrane fluidity and activation of protein kinase cAMP-dependent (PKA), protein kinase B (PKB), and protein kinase C (PKC) signaling pathways in rainbow trout hepatocytes (20, 21). In addition, this cortisol action also induces the extracellular signal-regulated kinase (ERK)—cAMP-responsive element binding protein (CREB) signaling pathways activation and the up regulation of peroxisome proliferator-activated receptor γ co-activator 1 α (*pgc1a)* expression in isolated rainbow trout myotubes (22). Even though, the mechanisms involved in the non-genomic cortisol pathways are complex and diverses, the impact in the adaptive response in fish are far from clear (23). In addition, until know whether non-canonical cortisol actions contribute to the regulation of glucose-metabolism gene expression is unknown.

Gilthead sea bream (*Sparus aurata*) is a well-studied marine fish used as a model in diverse studies related to physiological acclimation to stress (24, 25). Exogenous cortisol administration in *S. aurata* has revealed key aspects of this hormone in the transcriptional regulation of osmoregulatory processes, immune responses, and metabolic acclimation (26–28). In this line, even though it seems clear that cortisol increases circulatory glucose levels through the induction of hepatic gluconeogenesis-related genes, the impact of non-genomic cortisol actions over this process has not been explored yet.

In the present study, the rapid effects of membrane-initiated cortisol actions on the regulation of glucose and transcription of key glucose metabolism-related genes were studies in *S. aurata*. For this propose, we performed an *in vivo* assay in which *S. aurata* juveniles were injected with cortisol or with the membrane impermeable cortisol analog, cortisol-BSA. After 1 and 6 h of each treatment, plasma cortisol levels were measured, together with several metabolites in plasma, liver, and skeletal muscle. Hepatic transcript levels of corticosteroids receptors, as well as key gluconeogenesis and glycolysis related genes were also measured. The results were discussed in relation to the possible contribution of membrane-initiated cortisol actions on the regulation of glucose metabolism in fish liver and skeletal muscle.

## Methodology

### Emulation of Acute Stress in *S. aurata* Through the Exogenous Administration of Cortisol and Cortisol-BSA

Immature gilthead seabream (*S. aurata*) (16.1 ± 0.2 g body mass, mean ± SEM, *n* = 48) were obtained from Servicios Centrales de Investigación en Cultivos Marinos (SCI-CM, CASEM, University of Cadiz, Puerto Real, Cádiz, Spain; Spanish Operational Code REGA ES11028000312). Animals were randomly distributed in four 80-L tanks (~2.5 kg m^−3^ density) in a flow-through system, under natural photoperiod and constant temperature (18–19°C). Fish were fed daily with commercial pellets (1% of total animal weight). After 10 days of acclimation, fish of the first and second tanks were intraperitoneally administered with 0.01028 μM of cortisol (Sigma, St. Luis, MO) and 0.01028 μM of hydrocortisone-3-CMO-BSA (cortisol-BSA) (US biological, USA) dissolved in DMSO 1X, PBS 1X, and NP 40 0.05%, respectively. Preliminary experiments demonstrated that injection and manipulation of fish did not induce endogenous cortisol release and also this dose was appropriate to reach plasma levels of cortisol (~150 ng/mL) similar to those of an acute stress in *S. aurata* (29, 30) (Supplementary Figure 1). Fish of the third and fourth groups were administered with vehicle solution (DMSO, PBS 1X, NP 40 0.05%) and BSA (0.00892 mg/kg), respectively. The experiment was performed using duplicate tanks for each group. After 1 and 6 h of each treatment, all fish were euthanized by an overdose of 2-phenoxyethanol (1 mL/L). Blood was collected from caudal vessels with ammonium-heparinized syringes (Sigma-Aldrich H6279, 25000 units in 3 mL of saline 0.6 % NaCl).

Plasma was separated from cells by centrifugation of blood (3 min, 10,000 × *g*, 4°C), snap frozen in liquid nitrogen and stored at −80°C until analysis. The spinal cord of the fish was sectioned, and liver were collected, placed into tubes with 10-volumes (v/w) of RNA*later*™ Soln (Invitrogen by Thermo Fisher Scientific), held for 24 h at 4°C and then stored at −20°C until total RNA isolation. In addition, liver and skeletal muscle were also excised, and the biopsies collected in microtubes were snap frozen in liquid nitrogen and stored at −80°C until the assay of metabolites content. The experiment complied with the EU directives for the protection of animals used for scientific purposes (2010/63/EU), the Spanish laws (law 32/2007 and RD 53/2013), and it was authorized by the Ethical Committee of the Universidad de Cádiz (Spain) for the use of laboratory animals and the Ethical Committee from the Andalusian Government (Junta de Andalucía reference number 28-04-15-241).

### Plasma and Tissue Parameters

Plasma cortisol levels were measured by EIA kit (Arbor assays) as previously described by Estensoro et al. (31) in this fish species. Plasma glucose and lactate levels were quantified with the following Spinreact kits (Barcelona, Spain): HK Ref. 1001200 and Lactate Ref. 1001330, adapted to 96-well microplates. For the analysis of tissue metabolites, frozen skeletal muscle and liver were homogenized by ultrasonic disruption and analyzed according to the protocol of Vargas-Chacoff et al. (32). Glycogen levels were measured according to the method of Keppler and Decker (33), in which the glucose obtained via glycogen breakdown (after subtracting the free glucose levels) is determined using the previously described commercial glucose kit.

### Enzymatic Activities Assays

Frozen liver and skeletal muscle portions were homogenized by ultrasonic disruption in 10 volumes of ice-cold homogenization buffer (50 mM imidazole, 1 mM 2-mercaptoethanol, 50 mM NaF, 4 mM EDTA, 0.5 mM phenylmethylsulfonyl fluoride (PMSF) and 250 mM sucrose; pH 7.5). The homogenate was centrifuged for 30 min at 3,220 × g and 4°C, and the supernatant stored at −80°C for further analysis. Enzyme activities of: Glycogen phosphorylase (GPt), Hexokinase (HK), Fructose 1,6-bisphosphatase (FBP), Lactate dehydrogenase (LDH), and Glucose 6-phosphate dehydrogenase (G6PDH) were determined as previously described for *Sparus aurata* (34) using a PowerWave™ 340 microplate spectrophotometer (Bio-Tek Instruments, Winooski, VT, USA). Reaction rates of enzymes were determined by changes in absorbance from the reduction of NAD(P)+ to NAD(P)H, measured at 340 nm and 37°C, during pre-established times (10–15 min). Activities were referenced against the protein content of homogenate (U mg prot−1).

### RNA Extraction and cDNA Synthesis

Total RNA from *S. aurata* liver was extracted using the NucleoSpin RNA kit (Macherey–Nagel) according to manufacturer's instructions. An on-column RNase-free DNase digestion was used for gDNA elimination. RNA integrity and quantification were evaluated using 2100 Bioanalyzer and the RNA 6000 Nano Kit (Agilent Technologies, Santa Clara, CA, USA), and Qubit® 2.0 Fluorometer (Life Technology, Carlsbad, CA, USA), respectively. Only RNA with RIN >8 was used for cDNA synthesis. Retro transcription was carried out with the qScript™ cDNA Synthesis Kit (Quanta BioSciences) using 500 ng of liver RNA as input.

### Real Time PCR

Real time PCR was performed by semi-quantitative fluorescence with a CFX Connect™ Real-Time PCR System (Bio-Rad Laboratories) in 96 white wells Hard-Shell® PCR plates covered with Microseal® “B” Seals (Bio-Rad). Briefly, each reaction mixture (in a volume of 10 μL) contained 0.5 μL of each specific forward and reverse primers (4 μM), 5 μL of PerfeCTa SYBR Green FastMixTM 2x (Quanta BioSciences) and 4 μL containing 10 ng of cDNA from the *S. aurata* liver.

Several calibration plots with different template concentrations in serial dilutions (from 10 ng to 100 fg) of input total RNA from liver had amplification efficiencies between 92.9 and 104.7%, for all primer pairs used. The PCR profile was as follows: 95°C, 10 min; [95°C, 15 s; 60°C, 30 s] ×40 cycles; and a melting curve [60–95°C, increasing 0.5°C every 5 s]. The melting curve was used to ensure that a single product was amplified and to verify the absence of primer-dimer artifacts. Results were normalized against beta actin (*actb*) and elongation factor 1a (*ef1a*) as a internal reference genes. Relative gene quantification was performed using the ΔΔCT method (35).

Candidate sequences corresponding to fructose-bisphosphate aldolase (*aldo*), glucose 6 phosphatase (*g6pc*), *pepck, actb*, and *ef1*α were available in NCBI database. Sequence of phosphoglycerate mutase 1 (*pgam1*) was obtained from available database belong to the *S. aurata* sequencing project (36). Primers were designed using Primer 3 available tool (http://frodo.wi.mit.edu/primer3/) and validated in NetPrimer (http://www.premierbiosoft.com/netprimer/) and Oligo analyzer 3.1 (https://www.idtdna.com/calc/analyzer) available online tools. Finally, primers of glucocorticoid (*gr1, gr2*) and mineralocorticoid (*mr*) receptors were obtained from Tsalafouta et al. (37). All primers sequences used in this study are listed in Table 1.

**Table 1:** Primers used in this study.

**Gene name**	**Gene ID**	**Sequence 5^′^−3^′^**	**% Eff**	***R*^**2**^**	**Tm (^**°**^C)**	**Amplicon (bp)**
*Beta actin*	actb	F: TCTTCCAGCCATCCTTCCTCG R: TGTTGGCATACAGGTCCTTACGG	92.9	0.999	60	108
*Elongation factor 1α*	ef1a	F: AGAGGCTGTCCCTGGTGA R: TGATGACCTGAGCGTTGAAG	101.6	0.999	60	100
*Glucocorticoid receptor 1*	gr1	F: GGTTCAGCAGCAGTTCCTC R: GGTCTTGGTCGCCTTTATCC	99.7	0.988	60	197
*Glucocorticoid receptor 2*	gr2	F: ATCGTCAAGAGGGAGGAGAAC R: TTGGTATCTGGTTGGTGATGA	99.5	0.998	60	187
*Mineralocorticoid receptor*	mr	F: CGCCTGGCTGGAAAGCAGATG R: GAGGTCAGGGGCAAAGTAGAGCAT	105.7	0.996	60	189
*Phosphoenolpyruvate carboxykinase*	pepck	F: TCACACTCACGGACTGG R: CAATGATGGGACACTGACC	99.8	0.999	60	111
*Fructose-bisphosphate aldolase*	aldo	F: AATGTCCTTGCCAGATACGC R: TATACAGCCGCCAGAACCTT	99.7	0.999	60	137
*Phosphoglycerate mutase 1*	pgam1	F: AGAGGGGAAAAGGGTGCT R: CAGGCTTCAGGTTCTTGTC	98.7	0.985	60	153
*Glucose 6 phosphatase*	*g6pc*	F: GACCAAGAACACAAGCACCA R: AACCAGAGTCGTCGGGATTA	97	0.991	60	166

### Statistical Analysis

Normality and homogeneity of variances were analyzed using the Kolmogorov-Smirnov's and the Levene's test, respectively. All data were analyzed using a one-way ANOVA with treatment as the factors of variance, followed by a Tukey's honestly significant difference (HSD) as a *post-hoc* test, using the Graph Prism 7.0 software (GraphPad Software, Inc., San Diego, CA). A probability level of *p* < 0.05 was used as a threshold to indicate statistical significance.

## Results

**Figure 1:**
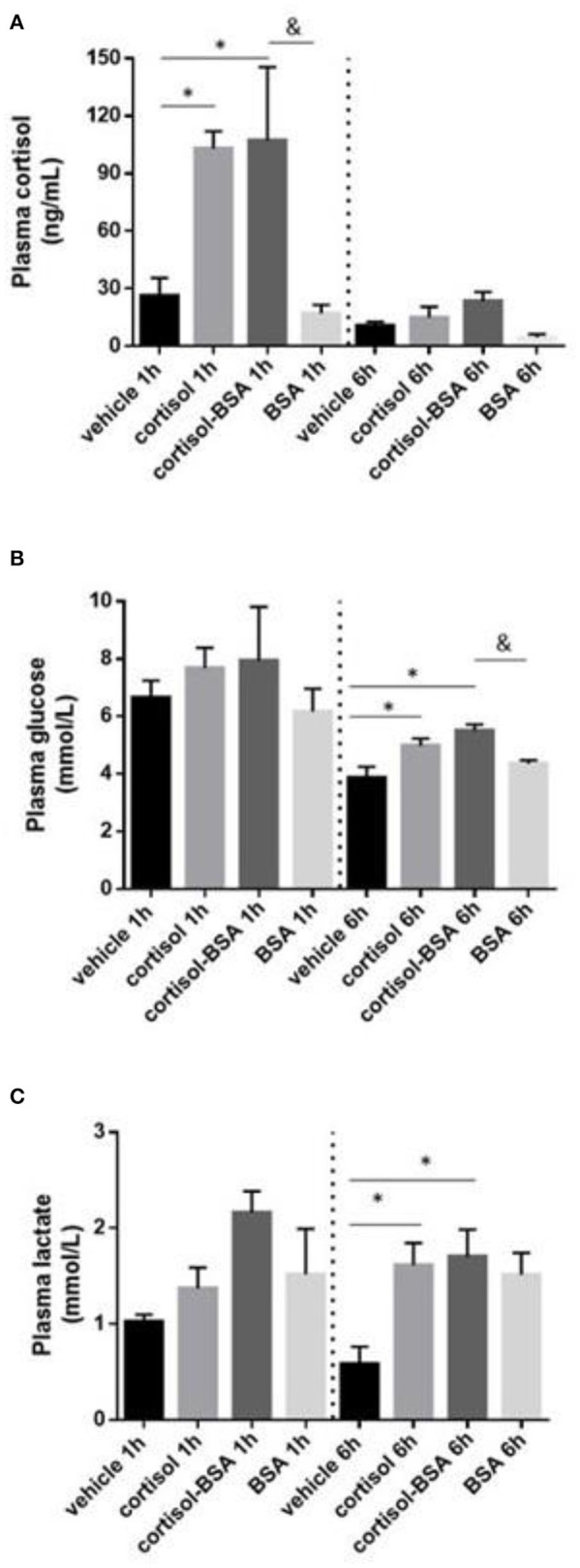
Plasma cortisol **(A)**, glucose **(B)**, and lactate **(C)** levels in vehicle (sham-control), cortisol, cortisol-BSA and BSA administrated fish at 1 and 6 h post-treatment. Results are expressed as mean ± SEM (*n* = 7). Asterisks and & represent significant differences (*p* < 0.05) against vehicle and BSA group at each sampling time, respectively.

*S. aurata* individuals intraperitoneally injected with cortisol and cortisol-BSA reached plasma cortisol levels of 103.0 ± 0.9 and 107.2 ± 38.1 ng/mL, respectively. However, these values returned to basal levels after 6 h of treatment (Figure 1A). In addition, plasma glucose and lactate levels enhanced after 6 h of cortisol and cortisol-BSA treatments (Figures 1B,C).

**Figure 2:**
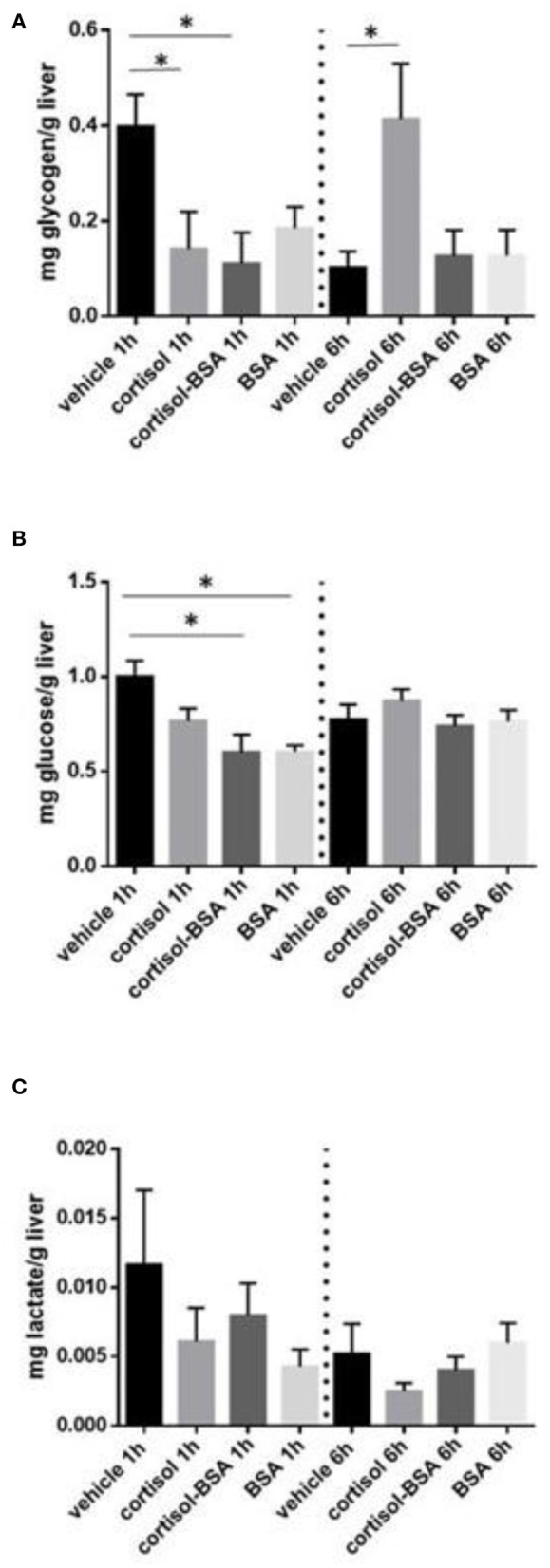
Hepatic glycogen **(A)**, glucose **(B)**, and lactate **(C)** levels in vehicle, cortisol, cortisol-BSA and BSA administrated fish at 1 and 6 h post-treatment. Results are expressed as means ± SEM (*n* = 7). Asterisks represent significant differences (*p* < 0.05) against vehicle group at each sampling time.

**Figure 3:**
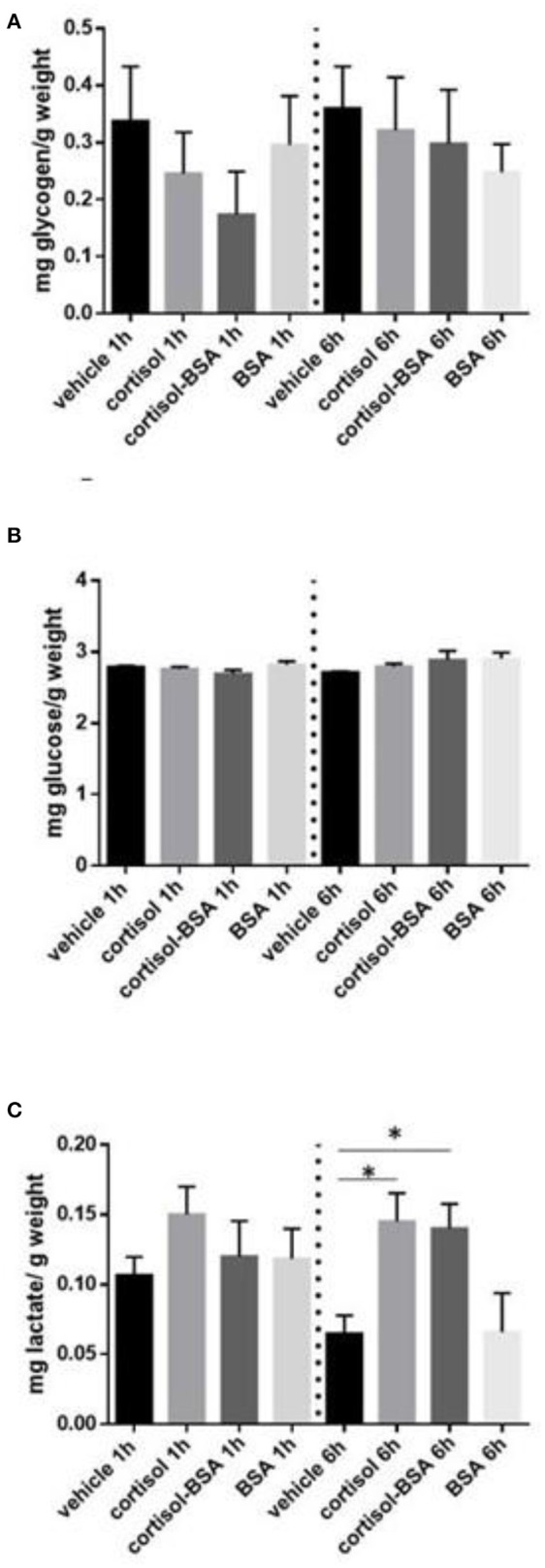
Skeletal muscle glycogen **(A)**, glucose **(B)** and lactate **(C)** levels in vehicle, cortisol, cortisol-BSA and BSA administrated fish at 1 and 6 h post-treatment. Results are expressed as means ± SEM (*n* = 7). Asterisks represent significant differences (*p* < 0.05) against vehicle group at each sampling time.

Liver glycogen content decreased after 1 h of cortisol and cortisol-BSA treatment (Figure 2A). However, these values were recovered in cortisol but not in cortisol-BSA group after 6 h of treatment (Figure 2A). Hepatic glucose levels decreased in both cortisol-BSA and BSA treated fish after 1 h (Figure 2B). Finally, there were no differences in liver lactate levels at 1 and 6 h of cortisol and cortisol-BSA treatment (Figure 2C). Regarding mobilization of energetic substrates in the skeletal muscle, there was no variation in glycogen or glucose levels in this tissue after cortisol treatment (Figures 3A,B). However, skeletal muscle lactate was increased at 6 h in both cortisol and cortisol-BSA treated *S. aurata* (Figure 3C).

To determine potentially effects of the membrane-initiated cortisol action on the regulation of glucose-metabolism related genes, we evaluated the activity of Glycogen phosphorylase (GPt), Hexokinase (HK), Fructose 1,6-bisphosphatase (FBP), Lactate dehydrogenase (LDH), and Glucose-6-phosphate dehydrogenase (G6PDH) in the liver and skeletal muscle. Overall, we observed that the activity of GPt, HK, and FBP does not change neither in the liver nor in skeletal muscle of injected *S.aurata* (Supplementary Figures 2A–C, 3A–C). Interestingly, we observed and increase in the activity of G6PDH in the liver of *S.aurata* treated with both cortisol or cortisol-BSA (Supplementary Figure 2E) and an increase of LDH activity in the skeletal muscle of *S.aurata* treated with cortisol-BSA (Supplementary Figure 3D).

To determine whether non-canonical cortisol effects contribute to modulate the expression of corticosteroids receptors and metabolism-related genes involved in the energetic substrate mobilization, we also evaluated hepatic mRNA levels of *gr1, gr2*, and *mr*, as well as key participants in gluconeogenesis (*g6pc* and *pepck*) and glycolysis (*aldo* and *pgam1*).

**Figure 4:**
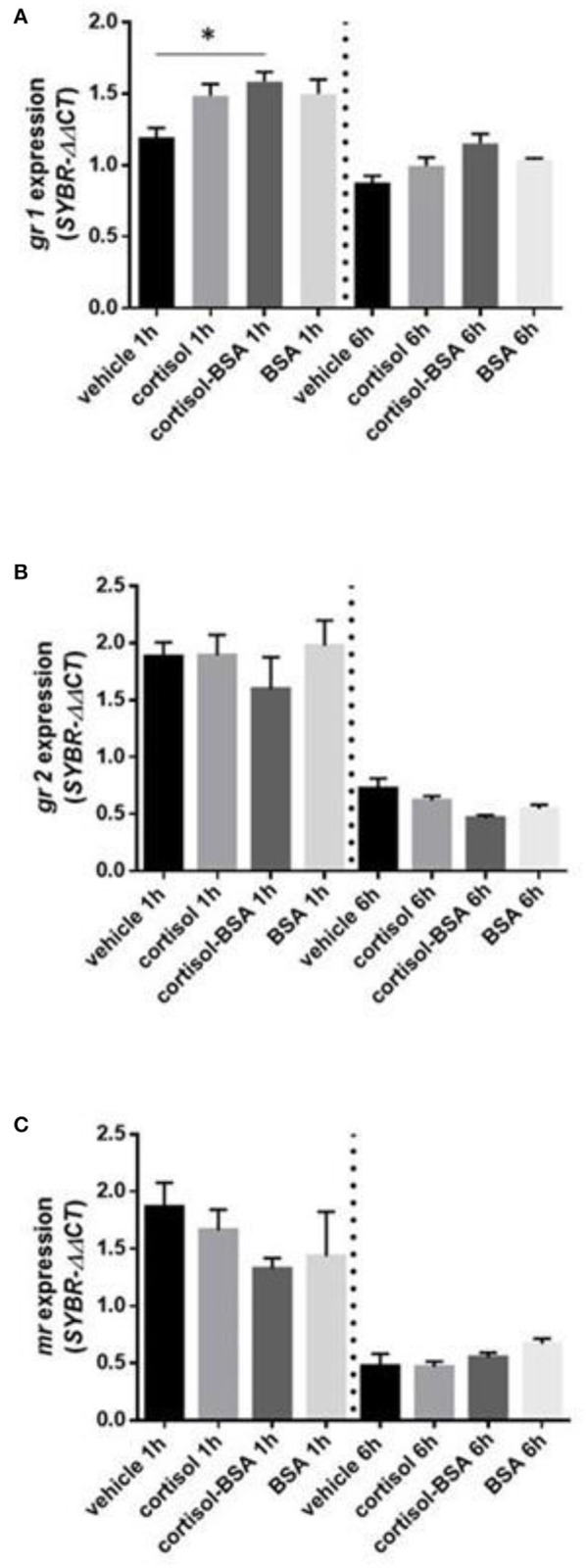
mRNA levels of corticosteroids receptor: glucocorticoid receptor 1 (*gr1*) **(A)**, glucocorticoid receptor 2 (*gr2*) **(B)** and mineralocorticoid receptor (*mr*) **(C)**. Expression values were normalized against elongation factor 1 alpha (*ef1*α) and beta actin (*actb*). Results are expressed as mean ± SEM (*n* = 6). Asterisks and & represent significant differences (*p* < 0.05) against vehicle and BSA group at each sampling time, respectively.

We observed a significant up regulation of *gr1* under 1 hour of cortisol-BSA treatment (Figure 4A). On the other hand, there was not change in *gr2* and *mr* expression in our experiment (Figures 4B,C).

**Figure 5:**
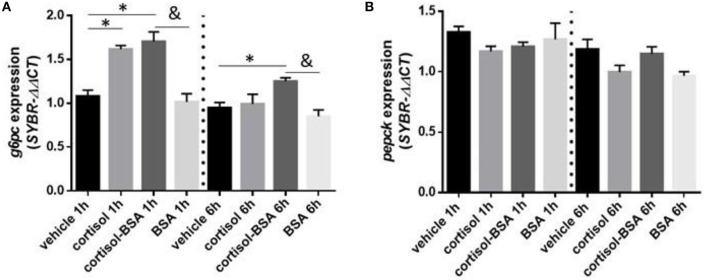
mRNA levels of hepatic gluconeogenesis enzymes glucose 6 phosphatase (*g6pc*) **(A)** and phosphoenolpyruvate carboxykinase (*pepck*) **(B)**. Expression values were normalized against elongation factor 1 alpha (*ef1*α) and beta actin (*actb*). Results are expressed as mean ± SEM (*n* = 6). Asterisks and & represent significant differences (*p* < 0.05) against vehicle and BSA group at each sampling time, respectively.

**Figure 6:**
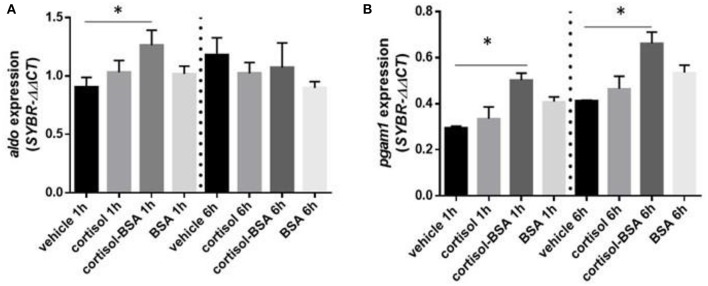
mRNA levels of hepatic glycolysis enzymes fructose-bisphosphate aldolase (*aldo*) **(A)** and phosphoglycerate mutase 1 (*pgam1*) **(B)**. Expression values were normalized against elongation factor 1 alpha (*ef1*α) and beta actin (*actb*). Results are expressed as mean ± SEM (*n* = 6). Asterisks and & represent significant differences (*p* < 0.05) against vehicle and BSA group at each sampling time, respectively.

Both cortisol and cortisol-BSA increased mRNA levels of *g6pc* at 1 h after treatment. However, cortisol-BSA, but not cortisol, enhanced this expression after 6 h (Figure 5A). Conversely, *pepck* mRNA levels remained without variations throughout the entire trial (Figure 5B). Both glycolysis-related genes assessed (*aldo* and *pgam1*) were up regulated at 1 h in the liver under cortisol-BSA but not cortisol treatment (Figures 6A,B). This enhancement was observed also at 6 h but only for *pgam1* expression (Figure 6B).

## Discussion

The stress response in fish is attributed to the genomic/classic mechanisms of action of cortisol, involving the interaction with intracellular GR and the subsequent modulation of stress-responsive genes (9). In this work, using the membrane-impermeable cortisol analog cortisol-BSA, we were able to provide evidence for those membrane-initiated cortisol actions contributing to the mobilization of energy substrates and the transcriptional regulation of glucose metabolism in *S. aurata*. These results are in line with our recently study that revealed that exogenous cortisol and cortisol-BSA administration is capable to emulate an acute stress condition in rainbow trout promoting the up-regulation of pyruvate dehydrogenase kinase 2 (*pdk2*) expression in skeletal muscle (38).

### Membrane-Initiated Cortisol Actions Modulate the Early Energy Substrates Mobilization in Liver and Skeletal Muscle of *S. aurata*

It is well-known that cortisol-mediated stress induces plasma glucose enhancement to provide energy for coping with stress (1, 4). In this work, we observed for the first time that stress-related doses of both cortisol and cortisol-BSA are able to increase plasma glucose levels in *S. aurata* 1 h after treatment. Because cortisol-BSA does not have the capacity to cross plasmatic membrane (38), our results suggest a potential role of membrane-initiated cortisol action in the glucose release to the bloodstream. In line with this result, recent studies using mammalian *in vitro* models have revealed that non-genomic cortisol pathways are involved in the rapid glucose mobilization, suggesting a possible role of this novel cortisol action in the cellular metabolic acclimation (39). In addition, this rapid non-genomic cortisol signaling has been associated with activity decrease of glucose related enzymes in fish, such glucose-6-phosphate dehydrogenase (G6PDH) and isocitrate dehydrogenase (ICDH) (40). Particularly, we observed and increase of G6PDH in the liver of *Sparus aurata* injected with cortisol or cortisol-BSA which support the idea that membrane-initiated cortisol action could be contributing in the glucose metabolism at this enzymatic level. However, the molecular mechanism underlying the glucose metabolism regulation owing to membrane-initiated cortisol action still is unclear. Interestingly, plasma glucose levels show a tendency to decrease in the vehicle group comparing 1 h with 6 h of treatment, this result could be related to a possible stress triggered by the handling of the animals and/or with the daily rhythmicity in the secretion of cortisol, which has previously been identified in *S.aurata* (41).

In addition to glucose, plasma lactate levels enhancement is another classic stress indicator that is also modulated by exogenous cortisol administration in fish (1, 28). We determined that plasma lactate levels also increased in *S. aurata* treated with cortisol and cortisol-BSA, suggesting a potential contribution to this non-canonical cortisol action in fish. However, until now, the non-canonical mechanisms of cortisol mediating increases of lactate are unknown.

A key metabolic response to stress is the hepatic metabolic capacity stimulation, promoting free glucose to coping with stress through hepatic gluconeogenesis as well as inhibition of glycolysis and glucose uptake in peripheral tissues (7, 42). Surprisingly, we observed that membrane-cortisol action is potentially involved in the mobilization of energy substrates in liver and skeletal muscle in *S. aurata*. Regarding the glucose regulation in the liver, we found that non-canonical cortisol effects induced glycogen breakdown (glycogenolysis) which is an essential process to provide free glucose to blood during the first phase of the stress response (26, 43). Even though glycogenolysis is mainly associated with the rapid secretion of catecholamines in teleost, including *S. aurata* (30, 44), *in vitro* mammalian models revealed that cortisol-related hormones (corticosterone) rapidly stimulates glycogenolysis by inducing the rapid phosphorylation of glycogen phosphorylase through a non-genomic mechanism (45). Therefore, we suggest that both classical intracellular membrane actions and rapid non-canonical glucocorticoid actions could be involved in the regulation of fish liver glycogenolysis. Moreover, it is well-known that cortisol is mainly associated to the replenishment of depleted glycogen stores after exposure to a stressor (4, 46). In this line, *S. aurata* individuals treated with cortisol, but not cortisol-BSA, increased hepatic glycogen content after 6 h, suggesting that glycogen content recovery is mainly mediated trough genomic cortisol actions. Once again, the variation observed in the hepatic glycogen levels between vehicle groups at 1 and 6 h support the idea of a possible handling stress in *S.aurata* caused during the *in vivo* protocol. Finally, we also observed a hepatic glucose content depletion in fish treated only with BSA, which could be attributed to the direct regulation of this compound (independent of the cortisol action) on the glucose metabolism in liver, similarly to the mammalian models (47). In this context, it has been previously reported that BSA alone could generates effects in different tissues potentially mediated by the osteonectin (SPARC) protein (48).

Regarding the role of non-genomic cortisol actions on energetic substrates mobilization in the skeletal muscle, *in vitro* studies revealed a direct role of glucocorticoids in the resynthesis of glycogen content in this tissue in rainbow trout (49). However, in the present study we did not observe changes in glycogen content either with cortisol or cortisol-BSA. It is not clear if this difference is due to species-specific differences or the outcome of *in vivo* versus *in vitro* approaches.

### Membrane-Initiated Cortisol Action Modulates the mRNA Levels of Glucose Metabolism-Related Genes in the Liver of *S. aurata*

There are several studies revealing the impact of cortisol on the regulation of genes involved in metabolic adaptation to stress (9, 28, 50). However, whether non-canonical cortisol actions present a role in the transcriptional response to stress are unclear (23).

The increase of *gr1* expression under 1 h of cortisol-BSA administration suggest that the rapid adjustments associated with short-term cortisol administration are modulated potentially by this receptor localizated in the surface. In line with this result, in other fish models has already propose the existence of a putative GR in the surface that mediated the rapid cortisol effects (38, 51). However, until now the presence a putative GR membrane receptor in *Sparus aurata* has not been identify yet. On the other hand, the variation of *gr2* and *mr* mRNA levels observed only between both experimental time (independent of exogenous administration of cortisol) could be associated with the daily rhythmicity on the cortisol secretion in *S.aurata* (41).

One of the surprising results of this work was to identify that *g6pc* is a potential target gene for membrane-initiated cortisol actions in the liver of *S. aurata*. G6Pase is a rate-limiting gluconeogenesis enzyme that has been defined as a direct cortisol target in vertebrates due to the presence of GRE sites in their promotor region (52). In addition, *in vitro* studies have revealed that cortisol directly up regulates *g6pc* expression in hepatocytes of rainbow trout after short-term stimulus (12). Therefore, we suggest that gluconeogenesis is possibly mediated trough membrane-initiated cortisol action in fish, specifically by increasing hepatic *g6pc* mRNA levels. Moreover, we did not find an increase in *pepck* mRNA levels, which represents another key rate-limiting enzyme of gluconeogenesis (53). Although *pepck* has been previously characterized as a target for the GR signaling in other teleosts such as rainbow trout (54), stress-induced cortisol does not necessarily increase hepatic *pepck* mRNA levels in fish (43).

Finally, part of the glucose production in the liver during the stress response is used in this tissue through glycolysis (43). In this matter, several glycolysis-related genes are up regulated under early stress events in the liver of fish (9, 55). In our study, mRNA levels of glycolysis-related genes (*pgam1* and *aldo*) of fish treated with cortisol-BSA enhanced at 1 and 6 h post-treatment, indicating a potential role of non-genomic cortisol actions in this process. These results suggest a major contribution of rapid non-genomic cortisol actions compared with the classical-initiated cortisol action, al least in the early regulation of glucose metabolism-related enzimes. However, this hypothesis needs to be tested and represents an interesting focus for futures studies.

## Conclusion

Taking all these results in consideration, we suggest that non-canonical cortisol actions contribute to the regulation of the early glucose metabolism response to stress in *S. aurata*. The precise mechanisms of this regulation are still unclear, but they would involve transcriptional regulation of key gluconeogenesis- and glycolysis-related genes such as *g6pc, pgam1*, and *aldo*.

## Data Availability Statement

The datasets generated for this study can be found in the Candidate sequences corresponding to fructosebisphosphate aldolase (aldo), glucose 6 phosphatase (g6pc), pepck, actb, and ef1α were available in NCBI database (https://www.ncbi.nlm.nih.gov/). Sequence of phosphoglycerate mutase 1 (pgam1) was obtained from available database belong to the S. aurata sequencing project (https://digital.csic.es/handle/10261/55459). Sequences obtained from NCBI: beta actin (actb), accession number: AY362763.1 elongation factor 1α (ef1α), accession number: AF184170.1 phosphoenolpyruvate carboxykinase (pepck), accession number: AF427868.1 fructose-bisphosphate aldolase (aldo), accession number: X82278.1 glucose 6 phosphatase (g6pc), accession number: AF427866.1 Sequence of phosphoglycerate mutase 1 (pgam1) obtained from *S. aurata* sequencing project: NCBI Sequence Read Archive SRA038178.1—http://hdl.handle.net/10261/55459.

## Ethics Statement

The animal study was reviewed and approved by the EU directives for the protection of animals used for scientific purposes (2010/63/EU), the Spanish laws (law 32/2007 and RD 53/2013), and it was authorized by the Ethical Committee of the Universidad de Cádiz (Spain) for the use of laboratory animals and the Ethical Committee from the Andalusian Government (Junta de Andalucía reference number 28-04-15-241).

## Author Contributions

JM, JV, and JA conceived and designed the study. JA and IR-J performed the experiments. JA, IR-J, and JM analyzed the data and performed statistical analyses. SB, AM, and GM-R contributed analysis, text, and comments to the paper. JA wrote the manuscript. All authors read and approved the final manuscript.

### Conflict of Interest

The authors declare that the research was conducted in the absence of any commercial or financial relationships that could be construed as a potential conflict of interest.
