# Supplementary material for: Contribution of Non-canonical Cortisol Actions in the Early Modulation of Glucose Metabolism of Gilthead Sea Bream (Sparus aurata)
Source: Front Endocrinol (Lausanne). 2019 Nov 12;10:779. doi: 10.3389/fendo.2019.00779 (PMC6863068; doi:10.3389/fendo.2019.00779)

Supplementary figure 1: Estimation of dose-response curve of cortisol in *S.aurata*. Fish were administrated with vehicle and 5, 2.5 and 1.25 mg of cortisol per kg of fish. After one hour, plasma was obtained from all fish and cortisol (A) glucose (B) and lactate was measured. Results are expressed as means ± SEM (n = 7). The *, *** and **** represent significant differences (p < 0.05, p < 0.005 and p < 0.001) against vehicle group at each sampling time, respectively.

Supplementary figure 2: Enzymatic activities of GPt (A), HK (B), FBP (C), LDH (D) and G6PDH (D); (U mg prot−1) in the liver of *S. aurata* after 1 h and 6 h of exogenous vehicle, cortisol, cortisol-BSA or BSA administration. Results are expressed as means ± SEM (n = 7). Asterisks represent significant differences (p < 0.05) against vehicle group at each sampling time.

Supplementary figure 3: Enzymatic activities of GPt (A), HK (B), FBP (C), LDH (D) and G6PDH (D); (U mg prot−1) in the skeletal muscle of *S. aurata* after 1 h and 6 h of exogenous vehicle, cortisol, cortisol-BSA or BSA administration. Results are expressed as means ± SEM (n = 7). Asterisks represent significant differences (p < 0.05) against vehicle group at each sampling time.

Supplementary figure 1

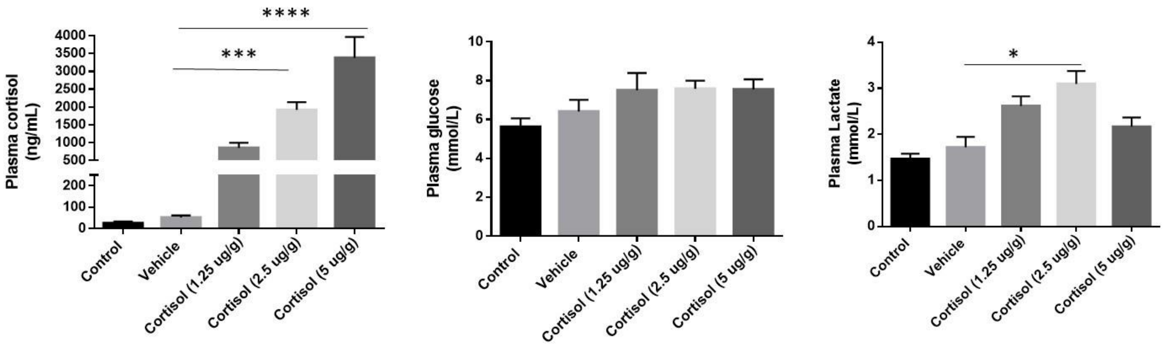

Supplementary figure 2

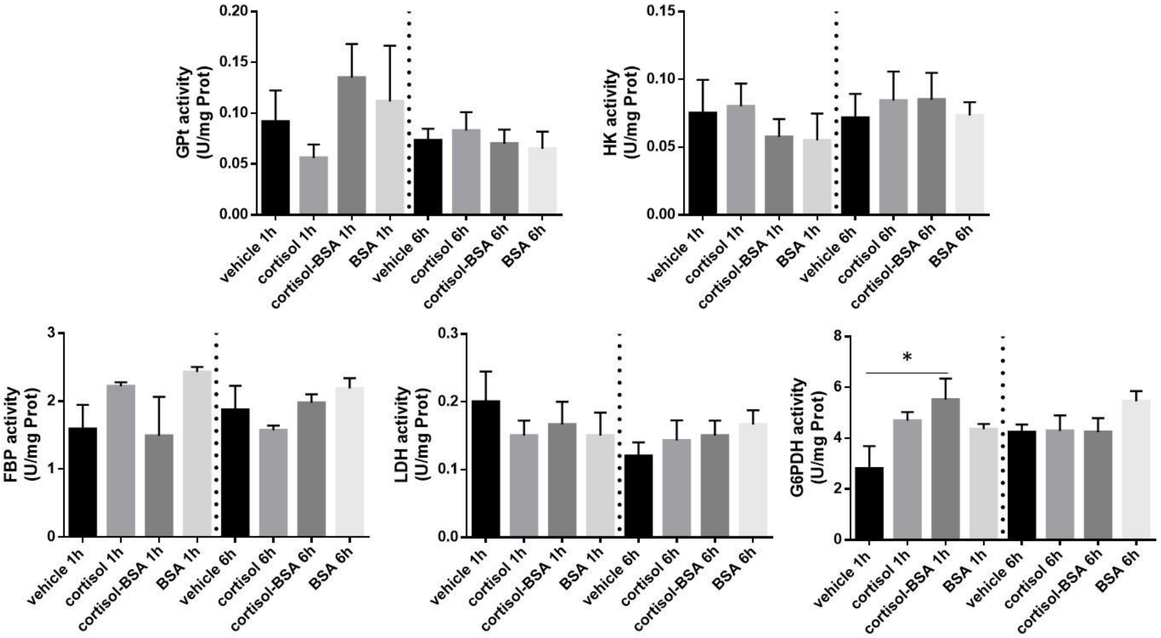

Supplementary figure 3

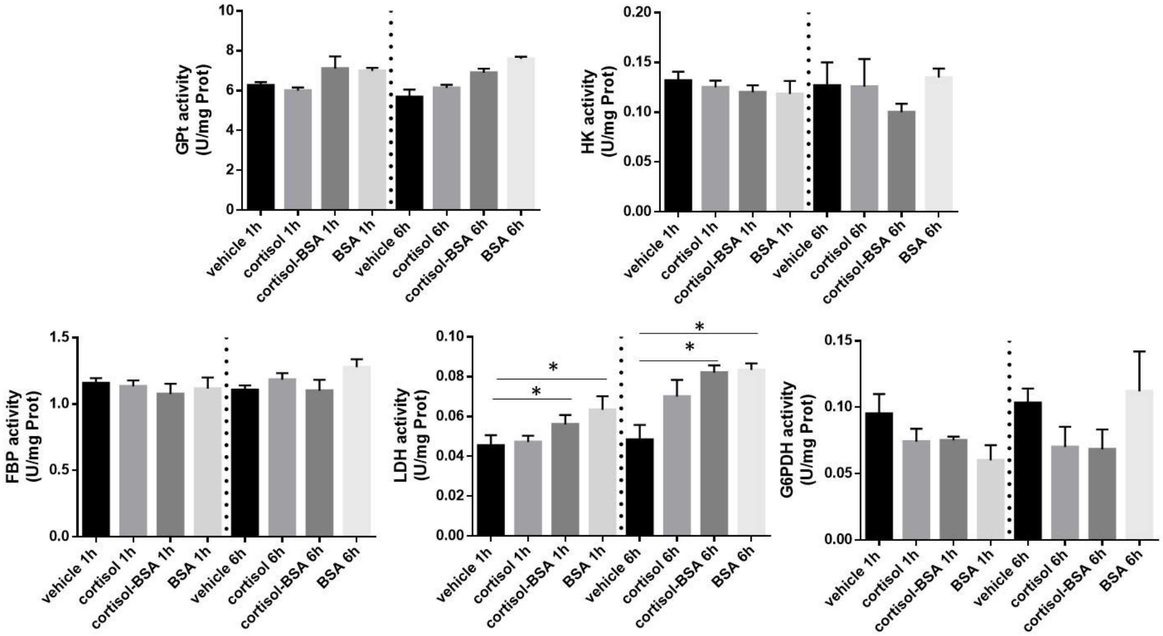

Supplement: Supplementary file 1 [file Data_Sheet_1.PDF]
